# Inadequate empirical antibiotics following debridement for orthopedic infections do not increase therapy failures

**DOI:** 10.5194/jbji-10-285-2025

**Published:** 2025-08-12

**Authors:** Steven Mark Maurer, Marc Simon Maurer, Marc Schmid, Stefani Dossi, Lucienne Gautier, Aileen Elizabeth Boyd, Mazda Farshad, Ilker Uçkay

**Affiliations:** 1 Department of Orthopedic Surgery, Balgrist University Hospital, Forchstrasse 340, 8008 Zurich, Switzerland; 2 Emergency Unit, Balgrist University Hospital, Forchstrasse 340, 8008 Zurich, Switzerland; 3 Unit of Clinical and Applied Research, Balgrist University Hospital, Forchstrasse 340, 8008 Zurich, Switzerland; 4 Infectiology, Balgrist University Hospital, University of Zurich, Forchstrasse 340, 8008 Zurich, Switzerland; 5 Department of Microbiology, King's College Hospital, Strand, London WC2R 2LS, United Kingdom

## Abstract

**Introduction**: Empirical antibiotics should only target the most likely pathogens if antibiotic stewardship is being heeded. However, there is a drive for broader-spectrum empirical antibiotics in orthopedic infections due to the concern of therapeutic failure if a regimen fails to target subsequently identified pathogens. **Methods**: Retrospective case-control study with surgically managed orthopedic infections from July 2018 to June 2024 with a minimum follow-up of 6 months. Patients were stratified by the initial empirical treatment of either accurate empirical choice or inaccurate empirical choice. **Results**: Of 482 infection episodes, 79 antibiotic regimens (43 broad-spectrum; 9 %) were used with a median postoperative duration of 42 d (interquartile range 19–45 d); 290 infection episodes (60 %) were correctly targeted. In 192 cases (40 %), the initial empirical choice was inaccurate, with a median switching time to a targeted treatment of 4 d. There was no difference between accurate and inaccurate empirical treatment in terms of ultimate failures (18/290 vs. 15/192; Pearson $\chi^{2}$ test, p=0.49 ), overall adverse events of therapy (15 % vs. 7 %, p=0.11 ), duration of hospital stay (median 9 d vs. 9 d, p=0.96 ), or supplementary surgical debridement (median 0 vs. 0 intervention, p=0.58 ). In multivariate logistic regression analysis, the duration of an inaccurate antibiotic treatment failed to alter the risk of “failures” (odds ratio 0.9, 95 % confidence interval 0.8–1.1). **Conclusions**: A delay in commencing targeted antibiotics does not increase the risk of a negative outcome. Narrower-spectrum empirical regimens are appropriate for clinically mild to moderate infections as a broader spectrum does not provide any clinical advantage.

## 1 Introduction

Antibiotic stewardship is of increasing importance in the 21st century, with international health authorities such as the World Health Organization (WHO) (Moja et al., 2024) and national scientific associations (Sendi et al., 2022; Uçkay et al., 2023a) promoting it in all healthcare disciplines. The feasibility of good antibiotic stewardship in orthopedic surgery (Feihl et al., 2022; Campbell et al., 2014; Uçkay et al., 2019; Kristensen et al., 2019) has been demonstrated, and groups in Switzerland have subsequently revised their guidelines regarding perioperative antibiotic prophylaxis (Sendi et al., 2022) in elective orthopedic surgery and open fractures (Uçkay et al., 2023a). They have investigated the shortening of total antibiotic administration (Uçkay et al., 2019; Nieuwland et al., 2023; Maurer et al., 2022) together with an early switch to oral medication (Nieuwland et al., 2023; Maurer et al., 2022; Sendi et al., 2023; Gallagher et al., 2023; Minotti et al., 2023) in stable patients. Adherence to revised guidelines and new recommendations should reduce drug and drug delivery costs and adverse events (Schindler et al., 2013a) and hopefully limit the spread of multiresistant pathogens.

There is sometimes resistance to the principles of antimicrobial stewardship in the choice of empirical antibiotics for orthopedic infections resulting in the use of increasingly broader-spectrum antibiotics. This may be due to concern about a polymicrobial infection or the presence of pathogens with either innate or acquired resistance to narrower-spectrum antibiotics (Veerman et al., 2022). It has been shown in vitro that biofilms are established within a few hours or days (Vandecasteele et al., 2004), so the surgeon may be concerned that any delay with targeted antibiotics may be associated with clinical failure.

There is a panoply of microbiological papers, especially from resource-poor settings, highlighting the issue of antibiotic resistance in orthopedic infections and recommending antibiotic treatment and prophylaxis (Veerman et al., 2022) accordingly. They overlook however whether such extensive empirical coverage is necessary for systemically well patients undergoing surgical debridement of a localized infection. Even short durations of unnecessary exposure to antibiotics, particularly broader-spectrum ones, can contribute to the selection (Friedl et al., 2024) and spread (Uçkay et al., 2023b) of multiresistant organisms and be associated with gastrointestinal side-effects (Schindler et al., 2013a) and renal or hepatic toxicity. There is therefore an argument to avoid broader-spectrum coverage and only treat the most likely virulent pathogens while awaiting the culture results 1–3 d later.

We explore this specific research question regarding the correct empirical antibiotic “coverage” in our tertiary center specializing in orthopedic infections. We hypothesize that an inaccurate empirical antibiotic regimen, or even omitting antibiotics until the results of the culture come in, would not harm patients, either in the first few days or in the months following surgery. Indeed, a pilot study at our institution in 2013 (Schindler et al., 2013b) and 2023 limited to diabetic-foot patients (Nieuwland et al., 2023) confirmed the safety of this approach. In contrast, we do not investigate surgical variables or patient co-morbidities in relation to therapeutic failures, for which a much broader literature is available.

## 2 Patients and methods

### 2.1 Setting and study definitions

Balgrist University Hospital is a tertiary referral orthopedic center in Switzerland. It has a large research center to investigate the management of orthopedic infections and has performed multiple retrospective and prospective trials of orthopedic infections since July 2018. This is a retrospective study of surgically managed orthopedic infections hospitalized between 2 July 2018 and 19 June 2024, with patients followed up for a minimum of 6 months. We excluded episodes with incomplete documentation, pediatric cases, direct admissions to the ICU (Davey and Marwick, 2008; Rhee et al., 2024), or cases without surgical debridement. In turn, the completeness of the individual surgical debridement, resections, the number of second looks, and other surgical parameters in the management of orthopedic infections were not the object of our study and are largely discussed in the corresponding literature. In this study, we merely focused on the empirical antibiotic choice after any debridement for infection.

The diagnosis of orthopedic infection was made based on clinical, radiological, histological, and/or microbiological criteria in deep-tissue samples that correspond to the most recent EBJIS (European Bone and Joint Infection Society; McNally et al., 2021) and IWGDF/IDSA (International Working Group on the Diabetic Foot/Infectious Diseases Society of America; Senneville et al., 2024) definitions and that are approved by our infection specialists. Basically, we defined infection as the presence of local inflammation and/or the presence of the same microorganisms in several deep-tissue and/or bone samples. Sonication results were facultative. Based on the pathogen identification and/or the corresponding antibiotic susceptibility testing, we assessed the accuracy of the choice of initial antibiotic therapy.

The Institute for Medical Microbiology at the University of Zurich performed all of the clinical bacteriological examinations according to the EUCAST criteria (Zbinden, 2013). Obviously, these new resistance testing results did not choose the initial empirical agent, but they enhanced the daily dose of the targeted antibiotic regimen with the new classification “susceptible, increased exposure” (Zbinden, 2013). Until recently, co-amoxiclav was the most frequently used empirical antibiotic agent for osteoarticular infections in Switzerland, and for this study we classified antibiotics as broad-spectrum if the spectrum surpassed co-amoxiclav. The following agents were considered broader-spectrum: ceftazidime, cefepime, piperacillin-tazobactam, carbapenems, daptomycin, linezolid, tigecycline, levofloxacin, systemic or local aminoglycosides, and glycopeptides. We defined “clinical failures” as the need for surgical revision or any new therapy at the former infection site for any reason, including for infection relapses. Conversely, “remission” was defined as the absence of any clinical, radiological, laboratory, or subjective suspicion of infection or the absence of evidence of “failure” after a minimum follow-up of 6 months.

An independent double-check of the key variables was conducted by two subgroups of investigators to detect discrepancies in the assessment of subjective data. In the case of discordance in data interpretation, the primary investigator was the final arbiter.

### 2.2 Study design and statistical analyses

This study necessitated a retrospective case-control design, as randomizing patients to receive inaccurate antibiotic therapy within a prospective trial would be ethically unacceptable. The Pearson $\chi^{2}$ test or Wilcoxon rank sum test were used to compare unmatched groups as appropriate. We stratified the initial empirical treatment into two major groups: accurate empirical choice (or targeted therapy from the start) and inaccurate empirical choice (with subsequent correction following the receipt of the intraoperative microbial results). We computed the delay until a correct antibiotic therapy was established as continuous (d) and categorized variables (groups ≤ 1 d, 2–5 d, and more than 5 d). The outcomes were therapeutic failures, length of hospital stay, number of surgical revisions, and adverse events in relation to the initial antibiotic regimen. To adjust for the case mix, we performed unconditional multivariate logistic regression analysis with the outcome “failure”, but only in logical and clinical relation to the antibiotic choice. The variables included in the final multivariate model are based on pre-existing scientific evidence and medically rational grounds. Likewise, we declined to include variables associated with general treatment failures after orthopedic surgery such as the surgical techniques or patients' co-morbidities. We checked for collinearity and effect modification by interaction terms and used the STATA^TM^ software (Version 15; College Station, USA). P values ≤ 0.05 (two-tailed) were significant.

## 3 Results

### 3.1 Infections and pathogens

**Figure 1:**
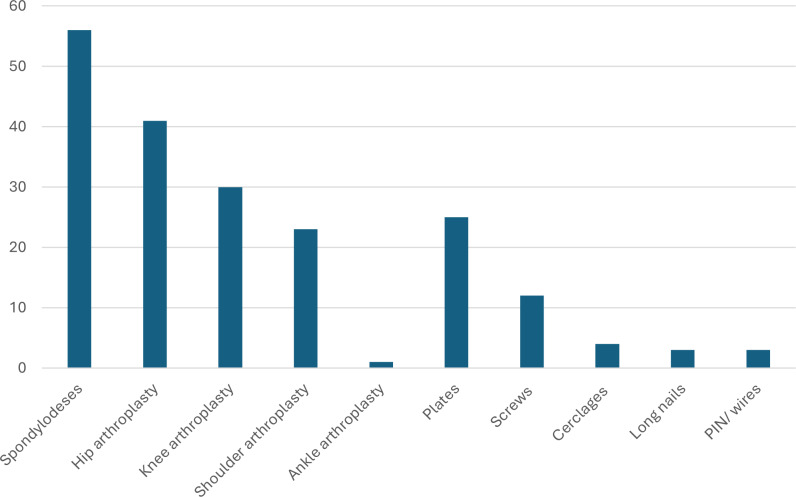
The most frequently infected implants in absolute numbers and descending order (from left to right).

Of the 482 independent orthopedic infection episodes, 136 (29 %) stem from diabetic-foot surgery, followed by spine ( n=89 ; 19 %), non-diabetic-foot (13 %), knee (11 %), shoulder (10 %), and hip (9 %) surgeries. Eleven infections involved multiple sites. The rest were distributed among hand, tumor, and sacral surgeries (paraplegia) with roughly 3 % each. Overall, 199 infections (41 %) were implant-related, 21 (4 %) native joint bacterial arthritis, 179 (37 %) implant-free osteomyelitis, and 74 (15 %) deep severe soft-tissue and/or tendon infections. Overall, 204 (42 %) were community-acquired infections. Figure 1 displays the most frequent implant types. In terms of causative pathogens, we noted 188 different microbiological constellations, of which *Staphylococcus aureus* represented one-third ( n=169 ; 35 %) of the causative pathogens. The group of skin commensals (coagulase-negative staphylococci, cutibacteria, micrococci, and corynebacteria) represented another third ( n=171 ; 36 %) and were mostly retrieved in implant-related cases. Of the 125 Gram-negative infections (26 %), 35 (7 %) were due to *Pseudomonas* spp. and 143 episodes (7 %) were polymicrobial. In polymicrobial cultures, the infectious disease physicians with experience in orthopedic infections determined clinically which pathogens caused the infections and which we should rather consider contaminants. This distinction was made before the analyses for this study.

### 3.2 Antibiotic regimens

Before surgery, 99 infections (21 %) were already under systemic antibiotic administration, mostly prescribed by general practitioners before admission; 79 % of the empirical antibiotic prescriptions were written in our clinic by the treating surgeons following consultation with at least 1 of 20 different infectious disease consultants. Postoperatively, excluding perioperative prophylactic agents, we used 79 different initial therapeutic antibiotic regimens in terms of agents, doses, and administration routes (not considering adaptations to the renal function). In total, the most frequently used empirical antibiotics were co-amoxiclav ( n=261 ), ciprofloxacin ( n=21 ), and clindamycin ( n=14 ). Forty-three regimens (43/482; 9 %) included a broad-spectrum agent at any time in the postsurgical period: vancomycin ( n=21 ), piperacillin/tazobactam ( n=14 ), levofloxacin ( n=5 ), cefepime ( n=1 ), ceftazidime ( n=1 ), and ertapenem ( n=1 ). In 101 episodes (21 %), there was no immediate postsurgical empirical therapy. In these latter cases, the clinicians introduced the antibiotic agents upon the first suspicion of positive intraoperative microbiological cultures. In our clinic, we do not use intraosseous (deep) local antimicrobial agents.

The high predominance in favor of co-amoxiclav (261/482; 54 %) was due to established recommendations by the Service of Infectious Diseases of Zurich University Hospital. The most frequently used perioperative prophylactic agent was parenteral cefuroxime (Sendi et al., 2022). The relatively high proportion of vancomycin and clindamycin among the empirical agents was partially due to the continuation of the perioperative prophylaxis as a therapeutic agent in patients with a (suspected) allergy to penicillin (Sendi et al., 2022). The median duration of the postoperative antibiotic regimen was 42 d (interquartile range 19–45 d) because the majority of orthopedic bone infections are still treated for 6 weeks according to international (and Swiss) recommendations (Rod-Fleury et al., 2011; Spellberg and Lipsky, 2012; Trampuz and Zimmerli, 2006). According to these recommendations, and by following the scientific literature on orthopedic infections, we treated skin commensals and/or low-virulence microorganisms the same way as pyogenic bacteria in terms of antibiotic duration (Uçkay et al., 2023b), except that their targeted treatment was broader in spectrum due to an inherent antibiotic resistance of many skin commensals.

In total, 32 patients (6.6 %) had a substantial adverse reaction to the antimicrobial treatment, necessitating a change or adjustment to the therapy. The most frequently recorded adverse events were diarrhea ( n=8 ), skin and vaginal mycoses (7), skin rash (5), and persisting nausea and gastric disturbances (4). Due to the rapid succession from empirical to targeted therapy and the retrospective nature of the analysis, we could not attribute the adverse events to the individual agents in question.

### 3.3 Accuracy of the empirical antibiotic regimen

Overall, 290 infection episodes (60 %) were correctly covered by the empirical antibiotic regimen according to the later microbiological cultures. Of these 290 episodes, the predominant pathogen was known before the first surgical debridement in 49 infections (10 %). Hence, in the 10 % of these episodes, the antibiotic treatment was labeled as targeted from the start. In 192 cases (40 %), the initial empirical choice was inaccurate, with mean and median times until switching to a correct (targeted) treatment of 8 and 4 d, respectively. There was no association with an inaccurate empirical choice, with demographic and clinical variables such as female sex, implant-related surgery, or diabetic-foot surgery. As expected, methicillin-susceptible *Staphylococcus aureus* significantly correlated with an appropriate empirical regimen ( p<0.01 ). In contrast, implant infections due to skin commensals ( p=0.04 ) and polymicrobial infections ( p<0.01 ) were significantly associated with an inaccurate initial regimen because the empirical antibiotics did not cover methicillin resistance or many additional pathogens.

### 3.4 Outcomes

We noted 34 therapeutic failures of antibiotic therapy of an infectious nature (34/482; 7 %) during or after the combined surgical and medical therapy. Table 1 compares patient groups with and without ultimate failures. Of note, there was no difference between the accuracy of the initial treatment and subsequent therapeutic failure reasonably related to the antibiotic therapy (18/290 vs. 15/192; $\chi^{2}$ test, p=0.49 ), adverse events (15 % vs. 7 %; p=0.11 ), hospital stay (median 9 d vs. 9 d, p=0.96 ), or the need for supplementary debridement (median 0 vs. 0 surgeries, p=0.58 ) (Table 1). Likewise, we did not encounter any new antimicrobial resistance among the pathogens of recurrent infection episodes. On purpose, we skipped to investigating the role of other important parameters such as underlying patient co-morbidities or the influence of various surgical interventions. Instead, these parameters, all without a rational link to the choice of the antibiotic agents, were investigated in group comparisons of “remission” and “failure” and showed no significant association with either outcome (Table 1).

**Table 1:** Comparison of episodes with and without treatment failures.

	Remission	$p^{b}$	Failure
	n=448		n=34
Female biological sex	158 (35 %)	0.49	10 (29 %)
Osteomyelitis without implant	169 (38 %)	0.40	10 (29 %)
Implant-related infection	186 (42 %)	0.71	13 (38 %)
– Total joint arthroplasties	86 (19 %)	0.26	9 (26 %)
Diabetic-foot surgery	130 (29 %)	0.68	11 (32 %)
Spondylodesis	52 (12 %)	0.93	4 (12 %)
Deep soft-tissue infections or abscesses	68 (15 %)	0.64	6 (18 %)
*S. aureus* infection	153 (34 %)	0.13	16 (47 %)
Gram-negative infection	116 (26 %)	0.94	9 (26 %)
– *Pseudomonas* spp.	31 (7 %)	0.29	4 (12 %)
Polymicrobial infection	133 (30 %)	0.97	10 (30 %)
Infection with skin commensals^a^	159 (35 %)	0.99	12 (35 %)
Correct empirical antibiotic coverage	106 (24 %)	0.99	8 (24 %)

^a^ Coagulase-negative staphylococci, cutibacteria, micrococci, and corynebacteria. ^b^ Pearson $\chi^{2}$ test.

### 3.5 Case-mix adjustment

Multivariate logistic regression analysis (Table 2) did not demonstrate any increased risk of “clinical failure” in patients receiving inaccurate empirical coverage. There was also no difference in clinical failure by duration of an inaccurate empirical regimen, whether computed as continuous (odds ratio 0.9, 95 % CI 0.8–1.1) or categorized variables (Table 2). Equally, the presence of an infected implant or diabetic-foot surgery did not alter that risk. The goodness-of-fit test was not significant ( p=0.13 ), and the receiver operating curve (ROC) value was 0.90, highlighting good accuracy of our final model despite the limited number of outcome variables.

**Table 2:** Logistic regression analysis with outcome failure (results expressed as the odds ratio with 95 % confidence intervals).

n=482	Univariate	Multivariate
	analysis	analysis
Female biological sex	0.8, 0.4–1.6	n.a.^a^
Implant-related infections	0.9, 0.4–1.8	0.8, 0.3–1.8
– Arthroplasties	1.6, 0.7–3.5	1.8, 0.7–4.7
– Spondylodesis	1.1, 0.4–3.1	1.1, 0.4–1.5
Diabetic-foot surgery	1.2, 0.6–2.5	n.a.
*S. aureus* infection	1.7, 0.8–1.3	n.a.
Infection due to skin commensals^b^	1.0, 0.5–2.0	n.a.
Gram-negative infection	1.0, 0.5–2.3	n.a.
Polymicrobial infection	1.0, 0.5–2.1	0.9, 0.4–2.1
Preoperative antibiotic use	1.0, 0.9–1.1	1.0, 0.9–1.1
Insufficient empirical coverage	0.9, 0.5–1.9	1.0, 0.2–2.4
Delay until correct therapy (continuous variable)	0.9, 0.8–1.1	0.9, 0.8–1.1
Delay until correct therapy (stratified analysis)		
– 1 d	1 (default)	1 (default)
– 2–5 d	1.4, 0.6–3.6	1.4, 0.4–3.7
– more than 5 d	0.4, 0.1–2.2	0.4, 0.1–2.6

^a^ n.a.: omitted from the final model because of the limited number of variables. ^b^ Coagulase-negative staphylococci, cutibacteria, micrococci, and corynebacteria.

## 4 Discussion

In this retrospective evaluation of 482 adult patients with a surgically managed orthopedic infection, 43 % received empirical antibiotic treatment that did not cover the pathogen(s) that were subsequently identified, whereas one-fourth of the study patients received antibiotics with unnecessarily broad coverage. Inadequacy of an antimicrobial regimen during the first few postoperative days did not result in increased failure of infection therapy, antibiotic-related adverse events, or extended hospital stay. It is possible that any deleterious effects of an initially inadequate regimen are reversed during the following 6 weeks of targeted therapy. Our study offers an in-depth view of the power of empirical antibiotic therapy in surgically managed orthopedic infections. The effect of the surgical (mechanical) debridement is likely many times higher than that of antibiotics, at least in the majority of patients with a local infection but no systemic compromise. We are confident that our findings are robust, because the antibiotic treatment of orthopedic infections relies on a few basic and common principles and is valid for all sorts of orthopedic infections, implants, and anatomical localizations. Additionally, we are unaware of any scientific evidence advocating in favor of an opposite experience. The 2 to 3 d of inadequate postsurgical coverage do not change the performance of the long and targeted antibiotic treatment. Consequently, and in the absence of clinical sepsis, we do not need to cover this empirically with broad-spectrum agents.

Our findings might not change the individual patient's infectious outcome, but they offer the possibility of enhancing antibiotic stewardship in orthopedic wards (Campbell et al., 2014). Broader-spectrum antibiotics can disrupt the patient's microbiota, leading to complications such as *Clostridioides difficile* colitis (Campbell et al., 2014; Schindler et al., 2013a), antibiotic resistance, and other drug-related side-effects (Gallagher et al., 2023; Schindler et al., 2013a). Moreover, in the context of rising healthcare costs, the financial implications of broader-spectrum empirical therapy cannot be ignored. Broader-spectrum empirical therapy, e.g., vancomycin, often involves the use of expensive antibiotics, intravenous drugs (Sendi et al., 2023; Gallagher et al., 2023), and extended hospital stays for therapy (Minotti et al., 2023) and its monitoring.

The scientific literature is very sparse regarding the significance of inaccurate empirical antibiotic regimens. We only know two similar articles. In 2013, we performed a similar pilot study in Geneva (Schindler et al., 2013b) with 342 cases of implant infections and followed them up for a median of 3.5 years. The median duration of empirical antibiotic coverage after surgical debridement was 3 d before switching to targeted therapy. Most empirical antibiotic regimens (269, 79 %) proved susceptible to the causative pathogen but were too broad in 111 episodes (32 %). Multivariate Cox regression analysis showed that neither susceptible antibiotic coverage (compared to non-susceptible antibiotic coverage; hazard ratio 0.7, 95 % confidence interval – CI – 0.4–1.2) nor broad-spectrum use (hazard ratios – HR – 1.1 and 0.8–1.5) changed the remission rates (Schindler et al., 2013b). Ten years later, Prost et al. (2023) investigated the outcomes of targeted and empirical antibiotic therapy in the treatment of adult patients with acute implant-free, community-acquired spondylodiscitis (Prost et al., 2023), and they concluded that there was no harm in waiting for the microbiological results for treatment in patients without neurological deficits or sepsis. Their study population differed from ours, and, importantly, only 38 % of their patients underwent surgical debridement (Prost et al., 2023).

We advocate abandoning the use of increasingly broad-spectrum empirical coverage in surgically managed stable patients, even if this lacks optimal coverage and even in the presence of implants. Instead, we advocate only targeting virulent staphylococci and streptococci (Kristensen et al., 2019; Minotti et al., 2023; Prost et al., 2023), an approach supported by the WHO (Moja et al., 2024). This is also supported by the findings of a study from the Mayo Clinic, Minnesota, USA, which demonstrated that culture-negative prosthetic joint infections can be treated successfully by empirical cefazolin alone with no excess of treatment failure (hazard ratio 0.7, 95 % CI 0.2–3.0) (Berbari et al., 2007), indicating that the main pathogens are only Gram-positive. We conclude that standard orthopedic infections are predominantly caused by methicillin-susceptible staphylococci (Minotti et al., 2023), streptococci, and (methicillin-resistant) skin commensals (Prost et al., 2023). However, severe soft-tissue infections of the ischemic diabetic foot and sacral decubitus ulcers may also involve anaerobes (Charles et al., 2015; Lebowitz et al., 2017), enterococci (Uçkay et al., 2017), and Gram-negative organisms, although these may be considered less virulent. The proportion of clinically relevant anaerobes (Lebowitz et al., 2017) and enterococci (Uçkay et al., 2017) in standard orthopedic infections remains low despite decades-long observation all over the world. The proportion of pyogenic Gram negatives is in a clear minority in Switzerland and other temperate regions (Uçkay and Bernard, 2010; Jamei et al., 2017) but might be higher in resource-poor settings in (sub)tropical regions (Uçkay and Bernard, 2010) or around the Mediterranean basin, where our findings may be less applicable.

Our study has many formal limitations: firstly, it is a single-center study in a temperate-zone, resource-rich country, which by nature limits the generalizability of the results on a global scale. Secondly, none of our patients was hemodynamically compromised or had rapidly spreading soft-tissue infections such as necrotizing fasciitis which would require a different clinical approach. Patients in the ICU and those with systemic infections require empirical broad-spectrum antibiotics as this increases the likelihood of appropriate therapy and might be life-saving (Davey and Marwick, 2008; Rhee et al., 2024). Thirdly, we cannot confidently state that any empirical antibiotics are needed in the immediate aftermath following debridement. Indeed, our own clinical experience and a retrospective German study involving approximatively a few dozens of operated spine infections suggest that antibiotics can be safely withheld until sufficient microbiological information is available for a narrow-spectrum targeted treatment (Prost et al., 2023). Fourthly, we cannot comment on the optimal duration of the targeted systemic antibiotic. In this study, the median duration of the postoperative antibiotic regimen was 42 d, in accordance with international (and Swiss) recommendations (Rod-Fleury et al., 2011) for 6-week treatment for orthopedic bone infections (Spellberg and Lipsky, 2012). Fifthly, we limit our study to the orthopedic discipline and focus entirely on the antibiotic part of infection therapy. Formally, our results cannot be extrapolated to other surgical parameters such as the completeness of debridement or be expanded to other surgical disciplines with much more heterogeneity of antibiotic treatment modalities such as in visceral surgery. Sixthly, diabetic foot infections especially are often polymicrobial and, unlike spine or native joint infections, it is frequently challenging to determine which cultured organisms are truly pathogenic and warrant treatment. In our database, the infectious diseases physician determined the pathogenicity of the various microorganisms in cultures. This arbitration, however, does not alter the objective fact of whether the initial empirical choice was formally correct or not. Lastly, our study would have been impossible if we had used local (intraosseous) antibiotics, which continue to represent the current research in reviews (Soldevila-Boixader et al., 2023) or prospective randomized trials (Dudareva et al., 2019). Local antibiotics might help to promote antibiotic stewardship, especially when combined with only a short course of systemic antibiotics. However, commercial local products are very often maximal in their antibiotic spectrum (e.g., consisting of vancomycin and/or gentamicin) and would theoretically cover all potential bacteria by default. Hence, with local antibiotics the empirical choice would formally always be correct, if not exaggerated.

## 5 Conclusion

A delay in commencing targeted antibiotics following surgical debridement for localized orthopedic infections does not increase the risk of therapeutic failures, adverse events, length of hospital stay, or the number of subsequent debridement procedures. We believe that amino-penicillins or first generation cephalosporins might be sufficient to bridge the short time until the availability of the microbiological susceptibility results. This narrow-spectrum empirical therapy followed by targeted therapy, based on the microbial antibiogram from the tissue taken at debridement, would contribute to better antibiotic stewardship in orthopedic wards and globally.

## Data Availability

Ilker Uçkay may provide anonymized data upon request. The data are not publicly available due to privacy concerns regarding protected health information.
